# Dr. Edward A. Fisher

**Published:** 1902-04

**Authors:** 


Dr. Edward A. Fisher, of Buffalo, died of apoplexy, March 1, 1902, aged 42 years. He was a district physician, a member of the staff of the Buffalo Homeopathic Hospital, and of the

Clinical Club. These organisations have taken appropriate action regarding Dr. Fisher's death and they attended the funeral in a body.

